# Case-Control Study of Platelet Glycoprotein Receptor Ib and IIb/IIIa Expression in Patients with Acute and Chronic Cerebrovascular Disease

**DOI:** 10.1371/journal.pone.0119810

**Published:** 2015-03-06

**Authors:** Peter Kraft, Christiane Drechsler, Ignaz Gunreben, Peter Ulrich Heuschmann, Christoph Kleinschnitz

**Affiliations:** 1 Department of Neurology, University Hospital Würzburg, Würzburg, Germany; 2 Department of Internal Medicine, University Hospital Würzburg, Würzburg, Germany; 3 Clinical Trial Center, University Hospital Würzburg, Würzburg, Germany; 4 Institute of Clinical Epidemiology and Biometry, Comprehensive Heart Failure Center, University of Würzburg, Würzburg, Germany; University of Muenster, GERMANY

## Abstract

**Background:**

Animal models have been instrumental in defining thrombus formation, including the role of platelet surface glycoprotein (GP) receptors, in acute ischemic stroke (AIS). However, the involvement of GP receptors in human ischemic stroke pathophysiology and their utility as biomarkers for ischemic stroke risk and severity requires elucidation.

**Aims:**

To determine whether platelet GPIb and GPIIb/IIIa receptors are differentially expressed in patients with AIS and chronic cerebrovascular disease (CCD) compared with healthy volunteers (HV) and to identify predictors of GPIb and GPIIb/IIIa expression.

**Methods:**

This was a case—control study of 116 patients with AIS or transient ischemic attack (TIA), 117 patients with CCD, and 104 HV who were enrolled at our University hospital from 2010 to 2013. Blood sampling was performed once in the CCD and HV groups, and at several time points in patients with AIS or TIA. Linear regression and analysis of variance were used to analyze correlations between platelet GPIb and GPIIb/IIIa receptor numbers and demographic and clinical parameters.

**Results:**

GPIb and GPIIb/IIIa receptor numbers did not significantly differ between the AIS, CCD, and HV groups. GPIb receptor expression level correlated significantly with the magnitude of GPIIb/IIIa receptor expression and the neutrophil count. In contrast, GPIIb/IIIa receptor numbers were not associated with peripheral immune-cell sub-population counts. C-reactive protein was an independent predictor of GPIIb/IIIa (not GPIb) receptor numbers.

**Conclusions:**

Platelet GPIb and GPIIb/IIIa receptor numbers did not distinguish between patient or control groups in this study, negating their potential use as a biomarker for predicting stroke risk.

## Introduction

The crucial contribution of platelets to lesion development after ischemic stroke (IS) has gained widespread acceptance [1,2], although their specific pathophysiologic role and interactions with endothelial or immune cells have not been wholly delineated. With the aid of transgenic mouse models and specific antibodies, extensive studies in the area of experimental stroke research have proved successful in distinguishing the pathways involved in pathologic thrombus formation [2,3].

In conventional terms, arterial thrombus formation can be considered to be a dynamic and multistep process, whereby platelets initially flow over vascular lesions, decelerate and subsequently ‘tether’ to the damaged vascular endothelium as a result of interactions with endothelium-derived von Willebrand factor (VWF) and the platelet-specific glycoprotein (GP) Ib-V–IX receptor complex [2,3]. In consequent steps, platelet GPVI receptors bind to sub-endothelial collagen while simultaneously activating platelets that, in turn, produce a conformational change in the GPIIb/IIIa receptors. Finally, activated GPIIb/IIIa receptors promote platelet aggregation by binding fibrinogen, which acts as a substrate for recruitment of additional platelets to the lesion site [2,3].

Evidence shows that inhibition of GPIb and GPVI receptors by antibodies or antibody fragments protects against IS formation in mice models of transient middle cerebral artery occlusion, without elevating the risk of bleeding complications [4,5]. Conversely, blockade of the final step of thrombus formation with monoclonal GPIIb/IIIa antibodies has not been shown to protect animals from IS and leads instead to increased rates of cerebral hemorrhage [4]. The observation that GPIb receptors not only stimulate thrombus formation, but also mediate inflammatory processes [6,7] may provide a rational explanation for the high efficacy seen in experimental stroke models of GPIb blockade.

In contrast to non-clinical models, there is limited knowledge surrounding the role and pathophysiologic relevance of GPIb and GPIIb/IIIa receptors in human stroke development. At best, a series of publications has focused on distinct platelet GP polymorphisms and their role as risk factors for vascular diseases, but the results remain controversial. It has been shown that the Kozak dimorphism of GPIb, but not the human platelet antigen (HPA)-Ib polymorphism, was associated with an increased risk of IS [8,9]. Another study reported that GPIb receptor numbers were elevated in patients with post-stroke depression [10]. Nevertheless, to date, the fundamental importance of GPIb and GPIIb/IIIa receptors in the clinical setting has been rarely investigated beyond genetic polymorphisms, and the factors that influence the expression of these GP receptor sub-types warrant identification. As the main ligand of the GPIb receptor, VWF is differentially regulated in patients with acute ischemic stroke (AIS)/transient ischemic attack (TIA) and chronic cerebrovascular diseases (CCD), as well as in healthy volunteers (HV) [11], whereas the regulation of GPIb and GPIIb/IIIa receptors has not yet been assessed in detail.

Additionally, despite experimental evidence that platelets have an important role in atherosclerotic plaque pathophysiology [12,13] very few studies have specifically examined the regulation of the GPIb and GPIIb/IIIa receptors in patients with CCD [14–16].

### Aims

This case—control study was conducted to evaluate whether GPIb and GPIIb/IIIa receptor numbers per single platelet differ between patients with acute cerebrovascular events (AIS/TIA) and those with CCD, and to identify demographic and clinical factors associated with GPIb and GPIIb/IIIa receptor counts.

## Methods

### Data collection

Patients with acute cerebrovascular events (AIS/TIA) and CCD were included, as were HV from the local population who were used as controls in the study. All patients and subjects participating in the study met the following inclusion criteria: blood withdrawal within 24 hours after symptom onset in patients with AIS (defined as acute ischemic lesion on brain imaging) and TIA (no acute lesion); presentation with extracranial and/or intracranial stenosis of the large cerebral arteries with (*n* = 66) or without (*n* = 51) a history of AIS or TIA for the CCD group; and age ≥ 50 years, no history of stroke, myocardial infarction, or peripheral arterial disease for the HV group. Patients with intracerebral hemorrhage, age <18 years, known plasmatic coagulation disorder or platelet dysfunction based on a detailed medical history were excluded from the study.

Study participants were consecutively recruited from the Stroke Unit (inpatients diagnosed with TIA or AIS), the outpatient department for CCD, or from a convenient sample of the local population (HV) after responding to poster requests at the Neurology Department, University Hospital of Würzburg, Germany, between September 2010 and January 2013. Informed written consent was obtained from all participants. The study protocol was approved by the ethics committee of the medical faculty of the University of Würzburg, Germany (reference number 65/2010). Overall, 116 patients with AIS or TIA, 117 patients with CCD, and 104 HV were eligible to participate in the study. Study participation did not affect treatment and patient care.

TOAST (Trial of Org 10172 in Acute Stroke Treatment) criteria [17] were applied to patients with AIS or TIA in an adapted format: (1) cardioembolism; (2) large-artery atherosclerosis; (3) small-vessel occlusion; or (4) other determined or undetermined etiology. Moreover, the National Institutes of Health Stroke Scale (NIHSS) [18] and Barthel Index score [19] were documented on patient admission, in addition to data on the interval between symptom onset and blood withdrawal, platelet inhibitor pretreatment, and acute stroke therapy modality (thrombolysis vs. no thrombolysis).

Cerebral lesion volume was measured using MIPAV software (medical image processing, analysis and visualization, Bethesda, US) on the basis of routine diffusion-weighted images (DWI) in a blinded fashion.

### Blood collection and measurements

Blood was drawn for sampling from an antecubital vein between 08.00 and 12.00 hours on day 0, 1, and 3 in the acute TIA or stroke group, and once in patients with CCD and HV using a 21-gauge butterfly needle. Specific standard operating procedures were followed for pre-analytic preparations for blood collection. Only non-hemolyzed blood samples were analyzed. The number of GPIb and GPIIb/IIIa receptors per single platelet was measured using a commercially available fluorescent-activated cell sorting (FACS) kit (Biocytex, ID 7004, Marseille, France) according to manufacturer’s instructions. After the staining procedure, platelet receptors were quantified by FACS analysis (FACSCalibur, Becton Dickinson, Heidelberg, Germany) using beads that enabled quantification of the distinct number of receptors per single platelet based on mean fluorescence intensity. Differential hematology parameters and C-reactive protein (CRP, measured in the AIS/TIA group) were analyzed at the Division of Laboratory Medicine of the University Hospital Würzburg.

### Statistical analysis

Continuous variables are expressed as mean with standard deviation or median with interquartile range (as appropriate), whereas categorical variables are presented as percentages. The association between the number of GPIb or GPIIb/IIIa receptors and demographic and clinical characteristics (age, sex, neurologic scales, disease modality [TIA or AIS], TOAST criteria, duration between symptom onset and blood withdrawal, NIHSS, Barthel Index, treatment modality [intravenous thrombolysis or not], and intake of platelet inhibitors in the days before blood withdrawal) was explored using analysis of variance (ANOVA) and the chi-square test. *P* values for comparisons across groups of clinical and demographic characteristics were derived from the aforementioned analyses, as appropriate. In order to identify potential predictors of GPIb and GPIIb/IIIa receptor numbers, a linear regression model was applied that included all variables without collinearity in a multivariate model with adjustment for age and sex. Coefficients and corresponding 95% confidence intervals were estimated using the model. GPIb and GPIIb/IIIa receptor numbers were compared between the different patient groups (AIS/TIA inpatients, CCD outpatients, or HV), and distributions analyzed using the Kolmogorov—Smirnov test. Numbers of GPIb and GPIIb/IIIa receptors were assumed to form a normal distribution, which were compared using ANOVA with a Bonferroni *post-hoc* test and additionally adjusted for age and sex. All reported *P* values are two-sided, with a *P* value <0.05 considered to be statistically significant. Analyses were performed using SPSS Version 21 and SAS software version 9.1 (SAS Institute Inc., Cary, NC).

## Results

### Descriptive analysis of the patients with an acute cerebrovascular event

Overall, 116 patients with AIS/TIA were included in the study, with a mean age of 70 ± 12 years and a predominance of males (53%). In patients with AIS or TIA, the baseline clinical severity measured with NIHSS and Barthel Index was 4.8 ± 6.0 and 74 ± 30, respectively. More than half of the patients had an AIS (58%). The demographic and clinical characteristics of patients with acute cerebrovascular events are summarized in Table 1.

**Table 1:** Baseline characteristics of patients with acute ischemic stroke/transient ischemic attack.

Characteristic	Value (*n* = 116)
Age, years	70 ± 12
Sex, *n* (%)	
Male	62 (53)
Female	54 (47)
Modality, *n* (%)	
AIS	67 (58)
TIA	49 (42)
TOAST criteria, *n* (%)	
Cardioembolism	70 (60)
Large-artery atherosclerosis	4 (3)
Small-vessel occlusion	12 (10)
Other determined or undetermined etiology	30 (26)
Thrombolysis, *n* (%)	34 (29)
Comorbidities, *n* (%)	
Hypertension	105 (91)
Diabetes mellitus	41 (35)
Hyperlipidemia	80 (69)
Renal failure	10 (9)
Atrial fibrillation	37 (32)
Persistent foramen ovale	28 (24)
Heart failure	5 (4)
Coronary artery disease	8 (7)
Family history of stroke	11 (9)
Smoking, *n* (%)	18 (16)
Platelet inhibitor before blood withdrawal, *n* (%)	87 (75)
Anticoagulation before blood withdrawal, *n* (%)	8 (7)
Lipid-lowering drug before blood withdrawal, *n* (%)	36 (31)
National Institutes of Health Stroke Scale at admission	4.8 ± 6.0
Barthel Index at admission	74 ± 30
Body mass index, kg/m^2^	27 ± 5
HbA_1c_, mmol/mol hemoglobin	46 ± 13
Lipid profile, mmol/l	
Total cholesterol	202 ± 52
Low-density lipoprotein	121 ± 45
High-density lipoprotein	51 ± 15
Triglycerides	157 ± 153
Duration between symptom onset and blood withdrawal, h	14 ± 7

AIS, acute ischemic stroke; HbA_1c_, glycated hemoglobin; TIA, transient ischemic stroke; TOAST, Trial of Org 10172 in Acute Stroke Treatment.

### Comparison of GPIb and GPIIb/IIIa receptor numbers in patients with AIS/TIA, CCD, and HV

Platelet-expressed GPIb and GPIIb/IIIa receptor numbers in patients with AIS/TIA and CCD compared with HV subjects are shown in Table 2. Primary analyses of data—that is, without adjustment for confounding factors—revealed significantly higher GPIb receptor numbers in patients with CCD (44078 ± 9492) compared with the HV (40965 ± 5890, *P* < 0.01) and AIS/TIA (41060 ± 6318, *P* < 0.01) groups, but this difference was eliminated after adjustment for age and sex in multivariate analysis (Table 2). As observed for GPIb, GPIIb/IIIa receptor numbers did not differ between any of the groups investigated (*P* = 0.87) (Table 2).

**Table 2:** Number of GPIb and GPIIb/IIIa receptors in patients with acute ischemic stroke/transient ischemic attack, as well as patients with chronic cerebrovascular disease and healthy volunteers by univariate and multivariate analysis.

	GPIb receptor numbers (mean ± SD)		GPIIb/IIIa receptor numbers (mean ± SD)
HV		40965 ± 5890	60807 ± 8125
CCD		44078 ± 9492	62368 ± 11233
AIS/TIA (day 0)		41060 ± 6318	61168 ± 9600
*P* (ANOVA, univariate analysis)		< 0.01	0.46
*P* (multivariate analysis, adjusted for age and sex)		0.87	0.87

AIS, acute ischemic stroke; ANOVA, analysis of variance; CCD, chronic cerebrovascular disease; GP, glycoprotein; HV, healthy volunteers; SD, standard deviation; TIA, transient ischemic stroke.

### Relationship between GPIb and GPIIb/IIIa receptor numbers and key demographic and clinical parameters in patients with an acute cerebrovascular event

In correlation analyses, the number of GPIb receptors was significantly associated with GPIIb/IIIa receptor numbers (*r* = 0.44, *P* < 0.001) and neutrophil counts (*r* = 0.23, *P* = 0.01). GPIIb/IIIa receptor expression was not associated with peripheral immune-cell sub-populations. In univariate analysis, neither GPIb nor GPIIb/IIIa receptor numbers correlated with CRP levels (*P* > 0.15). There was no correlation between cerebral lesion volume and GPIb (*r* = -0.15, *P* = 0.26) or GPIIb/IIIa receptor numbers (r = -0.06, P = 0.67) on day 0, respectively (data not shown).

Results from univariate analysis of the association between GPIb and GPIIb/IIIa receptor numbers and key demographic and clinical characteristics are summarized in Table 3. Only female sex displayed a trend towards association with GPIb receptor numbers (*P* = 0.06). GPIb and GPIIb/IIIa receptor counts per single platelet were not associated with patient age, disease (AIS vs. TIA), treatment modality (thrombolysis vs. no thrombolysis), etiology, severity of stroke (NIHSS, Barthel index), or antithrombotic treatment before stroke onset.

**Table 3:** Predictors of GPIb and GPIIb/IIIa receptor numbers in patients with acute ischemic stroke/transient ischemic attack by univariate analysis.

	GPIb (/platelet) (mean ± SD)	*P* value	GPIIb/IIIa (/platelet)(mean ± SD)	*P* value
Sex				
Male	42088 ± 6993		62163 ± 11150	
Female	39881 ± 5261	0.06	60027 ± 7376	0.23
Age, years				
<55	41010 ± 5002		61178 ± 8676	
55–64	43841 ± 8222		64660 ± 14165	
65–74	39887 ± 4889		60770 ± 7858	
75–84	40048 ± 6818		58131 ± 7194	
>84	41197 ± 4589	0.15	62387 ± 7294	0.17
Disease modality				
AIS	40927 ± 5457		60810 ± 10199	
TIA	41243 ± 7391	0.79	61659 ± 8796	0.64
Modified TOAST criteria				
Cardioembolism	41153 ± 5746		60410 ± 8294	
Large-artery atherosclerosis	41794 ± 2503		58952 ± 4799	
Small-vessel occlusion	41160 ± 6512		65874 ± 8463	
Other determined or undetermined etiology	40707 ± 7922	0.98	61352 ± 12688	0.32
Duration between symptom onset and blood withdrawal, h				
<5	40581 ± 5259		60971 ± 9062	
5–12	41188 ± 5886		65674 ± 14779	
12–24	41164 ± 6838	0.98	60494 ± 8407	0.21
National Institutes of Health Stroke Scale				
0–4	41288 ± 6861		60606 ± 8208	
5–9	40279 ± 4949		60838 ± 8160	
10–15	41585 ± 7271		67417 ± 17617	
>15	40331 ± 3134	0.90	58384 ± 8911	0.12
Barthel Index				
0–30	37717 ± 2864		63137 ± 6638	
35–70	40408 ± 6314		63452 ± 15579	
>70	41185 ± 6135	0.28	60509 ± 7801	0.52
Thrombolysis				
Yes	40760 ± 5660		92959 ± 12715	
No	41185 ± 6601	0.74	60426 ± 7942	0.20
Platelet inhibitor before blood withdrawal				
Yes	41330 ± 6543		60731 ± 10057	
No	40603 ± 5965	0.55	61912 ± 8834	0.52

AIS, acute ischemic stroke; GP, glycoprotein; TIA, transient ischemic stroke; TOAST = Trial of Org 10172 in Acute Stroke Treatment.

In the multivariate analysis (adjusted for age and sex), CRP was identified as an independent predictor of GPIIb/IIIa (*P* = 0.03) but not of GPIb (*P* = 0.73) receptor numbers. The trend towards an association between sex and GPIb receptor count was maintained in the multivariate analysis (*P* = 0.07). As observed for univariate analysis, other demographic and clinical parameters were not associated with GPIb or GPIIb/IIIa receptor numbers after multivariate adjustment (Table 4). Additionally, the time point of blood withdrawal (days 0, 1, and 3) did not influence GPIb (*P* = 0.77) and GPIIb/IIIa (*P* = 0.26) receptor numbers (data not shown).

**Table 4:** Predictors of GPIb and GPIIb/IIIa receptor numbers in patients with acute ischemic stroke/transient ischemic attack by multivariate analysis.

	GPIb				GPIIb/IIIa	
	Coefficient	95% CI	*P* value	Coefficient	95% CI	*P* value
Sex						
Male	Reference			Reference		
Female	−2277.71 ± 1257.54	–4772.02 to 216.61	0.07	−2122.88 ± 1812.86	−5718.67 to 1472.91	0.24
Age, years			0.55			0.32
<55	Reference			Reference		
55–64	3603.24 ± 2172.36	−705.63 to 7912.11		4960.14 ± 3131.67	−1251.51 to 11171.79	
65–74	−538.87 ± 2026.53	−4558.49 to 3480.75		−9.03 ± 2921.44	−5803.69 to 5785.64	
75–84	−6.19 ± 2143.12	−4257.07 to 4244.68		−1076.71 ± 3089.51	−7204.75 to 5051.32	
>84	1633.88 ± 2720.51	−3762.23 to 7030.00		3007.42 ± 3921.87	−4771.59 to 10786.44	
Disease modality (TIA vs. AIS)	−783.93 ± 1326.76	−3415.55 to 1847.68	0.56	2463.00 ± 1912.65	−6256.73 to 1330.72	0.20
National Institutes of Health Stroke Scale			0.74			0.79
0–4	Reference			Reference		
5–9	−599.58 ± 1881.43	−4331.39 to 3132.23		−855.46 ± 2712.26	−6235.22 to 4524.31	
10–15	0.38 ± 2225.28	−4413.46 to 4414.21		5587.42 ± 3207.95	−775.54 to 11950.38	
>15	−1334.20 ± 2607.31	−6505.82 to 3837.42		−5456.87 ± 3758.71	−12912.25 to 1998.52	
Thrombolysis	224.31 ± 1631.59	−3011.93 to 3460.56	0.89	3274.15 ± 2352.09	−1391.20 to 7939.50	0.17
Use of platelet inhibitor before blood taking	321.05 ± 1327.00	−2311.05 to 2953.15	0.81	−1130.11 ± 1913.00	−4924.53 to 2664.31	0.56
C-reactive protein at admission, mg/dl	52.19 ± 152.11	−249.53 to 353.90	0.73	493.06 ± 219.28	58.11 to 928.01	0.03

AIS, acute ischemic stroke; CI, confidence interval; GP, glycoprotein; TIA, transient ischemic stroke.

## Discussion

To the best of our knowledge, no published study has systematically assessed GPIb and GPIIb/IIIa receptor numbers in patients with AIS/TIA and CCD. The findings from this case—control study showed that GPIb and GPIIb/IIIa platelet receptors are not differentially regulated in patients with AIS/TIA or in patients with CCD or in HV. Nevertheless, within the AIS/TIA group, inflammatory markers were positively correlated with GP receptor numbers (GPIb with neutrophils; GPIIb/IIIa with CRP), thus being indicative of an association between thrombus formation and inflammatory processes.

As observed with GPIb inhibition [4], blockade of VWF also leads to profound protection from IS in mice [20,21]. To evaluate the regulation of VWF in humans, we previously assessed serum levels in the population reported in this study and observed differential regulation of VWF, with higher levels in patients with CCD compared with HV, and patients with AIS/TIA having even higher levels than the CCD group [11]. Therefore, in this study, the finding that GPIb receptor numbers are unchanged between these patient groups is unexpected at first glance. However, a potential explanation for the lack of differentiation between GPIb receptor numbers is the postulated downregulation of GPIb receptors as a result of ectodomain shedding that is induced by platelet agonists during modulation of platelet function [22]. Indeed, a recent study of Chinese patients with atherosclerotic IS demonstrated lower expression levels of GPIb but higher levels of a disintegrin and metalloproteinase 17 (ADAM17), an enzyme that has been reported to play a major role in GPIb shedding [23].

In our study, GPIb and GPIIb/IIIa receptor numbers correlated with inflammatory processes such as neutrophil count and CRP levels. This relationship between platelet markers and inflammation is in accordance with our novel theory, which defines stroke as a thrombo-inflammatory disease rather than solely a thrombotic disease. Thrombo-inflammation is recognized as being an important mediator of IS pathophysiology in rodents [24–26], which, in broad terms, can be defined as the interaction of thrombotic (e.g. coagulation factors, platelets) and inflammatory (e.g. immune cells) processes at the ischemic neurovascular unit [6,7]. In IS, similar associations between thrombotic and inflammatory markers have also been described for plasma coagulation factors such as VWF [11] and factor XII [27].

To date, there have been few or no standardized assessments of GPIb and GPIIb/IIIa receptors in patients with CCD. Our findings of minimal variation in GPIb and GPIIb/IIIa receptor numbers in the CCD group versus the AIS/TIA group and HV (in multivariate analysis) does not preclude a pathophysiologic role for these platelet receptors. Nevertheless, recent publications suggest that the GPIb-V–IX receptor complex and GPIIb/IIIa receptors are not essential for the development of atherosclerotic lesions [14,15,28,29], although platelets are implicated in the early stages of atherosclerosis [29,30].

The limitations of this study require some consideration. It should be borne in mind that the potential for reverse causation induced by blood withdrawal post-cerebrovascular events cannot be disregarded. Thus, our study describes the degree and significance of associations between GPIb and GPIIb/IIIa receptor numbers and demographic/clinical parameters without assigning causality. Instead, prospective studies are warranted to address this limitation. Due to ethical constraints, which precluded the recruitment of individuals who were unable to provide informed consent, it may not have been possible to ensure that patients with very large strokes and/or aphasia were fully represented in our study. Moreover, a non-vascular origin for symptoms could not be completely discounted in 42% of the TIA patient population, and 7% of patients with AIS/TIA were administered anticoagulant treatment. Thus, there remains a possibility that the aforementioned factors may have influenced the regulation of GPIb and GPIIb/IIIa receptors.

## Conclusions

Results from this study did not show differential regulation of platelet GPIb and GPIIb/IIIa receptors in patients with AIS or CCD. Hence, our findings suggest that GPIb and GPIIb/IIIa receptor quantification is unlikely to represent a clinically valid method for delineating patients at risk of a cerebrovascular event.
